# Supplementary figures and images for: Genome wide analysis reveals heparan sulfate epimerase modulates TDP-43 proteinopathy
Source: PLoS Genet. 2019 Dec 13;15(12):e1008526. doi: 10.1371/journal.pgen.1008526 (PMC6934317; doi:10.1371/journal.pgen.1008526)

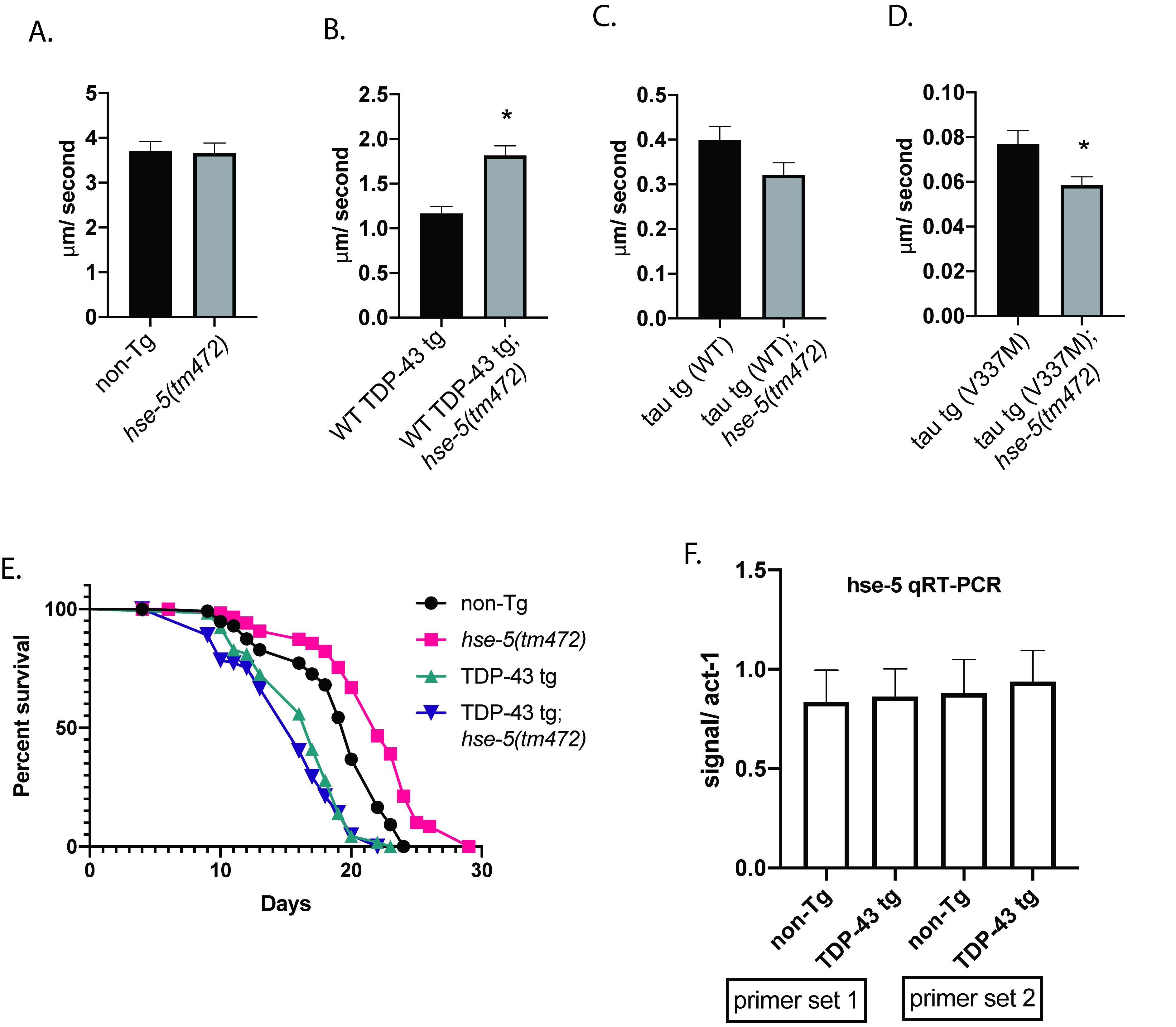

Supplement: S2 Fig — (A) hse-5(tm472) do not have significant differences in motility relative to non-transgenic (non-Tg) C. elegans, N>200 per genotype. (B) Motility defects of C. elegans expressing wild-type TDP-43 (WT TDP-43 tg) are significantly improved by hse-5(tm472). p<0.0001, unpaired t-test, N>200 per genotype. (C) hse-5(tm472) does not significantly alter the motility defects in C. elegans expressing wild-type human tau, although it is trending towards worsened motility. p = 0.052, unpaired t-test, N>130 per genotype. (D) hse-5(tm472) significantly worsens motility defects in C. elegans expressing mutant V337M human tau. p = 0.0097, unpaired t-test, N>150 per genotype. (E) hse-5(tm472) are significantly long-lived relative to non-Tg animals (N2), p<0.0001, Log-rank test. TDP-43 tg are short-lived relative to non-Tg animals, p<0.0001. However, the lifespan of TDP-43 tg; hse-5(tm472) are not significantly different from TDP-43 tg animals alone, p = 0.143. (F) mRNA expression of hse-5 does not change in TDP-43 tg relative to non-Tg animals. Two different primer pairs were tested using quantitative reverse transcription real-time PCR (qRT-PCR) on three independent replicate samples. Signal was normalized to the expression an internal reference gene, act-1. (TIF) [file pgen.1008526.s002.tif]
