# Supplementary material for: Genome wide analysis reveals heparan sulfate epimerase modulates TDP-43 proteinopathy
Source: PLoS Genet. 2019 Dec 13;15(12):e1008526. doi: 10.1371/journal.pgen.1008526 (PMC6934317; doi:10.1371/journal.pgen.1008526)
Supplement: Attachment — Submitted filename: Response to REviews_9_11_19_.pdf [file pgen.1008526.s003.pdf]

Reviewer #1:

1) For the Western blots of TDP-43 and pTDP-43, some quantification of the results would be helpful. The suppressor hits that did not change TDP-43 and pTDP-43 levels would also be interesting to see.

We have added quantification of the Western blot data in Figure 2, and we have also added Western blot data and quantification for the suppressor hits that did not change TDP-43 or pTDP-43 levels (Supplemental Figure 1C-F).

2) In regards to TDP-43 levels, the authors are careful to avoid saying that the decreased levels are due to improved clearance, but nonetheless it would be important to see if the candidates change TDP-43 mRNA levels. If they do so, it would not disqualify the candidates, but one would have a better, different idea of mechanism of protection.

We agree this is an important point. To address it, we have performed qRT-PCR to assess mRNA expression levels of the TDP-43 transgene, and found that none of the suppressor strains have decreased expression of the transgene. These data have been included as Supplemental Figure 1A-B.

3) In regards to hse-5, one wonders if adult only (or from L4 onwards) RNAi has an effect and would avoid the potentially protective/potentially harmful axonal abnormalities

We appreciate the Reviewer's suggested experiment, but struggle with the potential interpretations. The initiation of RNAi at later developmental stages typically leads to much weaker phenotypic effects. We believe it unlikely that we could draw clear conclusions from this experiment if there is no effect from late RNAi treatment. This likely outcome could either be caused by normal axon development or the very weak expected effect of L4 RNAi. It would be very difficult to distinguish between these two possibilities.

4) The screen was performed in a mutant TDP-43 line, however the cell culture and FTLD-TDP would presumably express wild-type TDP-43. Therefore, it would be interesting to see if the hse-5 RNAi or mutant is protective against the WT TDP-43.

We now include data showing that *hse-5(-)* protects against wild-type TDP-43 as well (Supplemental Figure 2B).

5) Also, Table 2 is referred to in the text, but was not included in the manuscript for review.

We thank reviewers for pointing out this absence. We apologize for this oversight and have included Table 2 with the resubmission materials.

Reviewer #2:
Major points
Unbiased genetic screens are powerful ways to advance understanding. However, sometimes it is not evident by which mechanism a modifier achieves its phenotypic effects. Here the authors sequentially limit their candidate genes to a select few before settling on hse-5/GLCE. There is no doubt that this gene has an effect on phosopho TDP-43 phenotypes. But the mechanism for the suppression is not sufficiently accounted for.
p. 10 "Preliminary characterization of GLCE suggests it can act to detoxify pTDP and is a therapeutic target worth further examination." The choice of focusing on one gene, hse-5 is reasonable, but I have several questions. How does a potential ECM protein affect the levels of TDP-43 phosphorylation in worms and tissues? What is the working model? The gap in understanding should be addressed, at least as speculation in the discussion.
We appreciate this suggestion and we have expanded our discussion of the potential protective mechanisms at work in *hse-5*/ GLCE loss of function . This includes discussion of the ability of ECM to interact and influence neuronal function, and in support of this, we have included new data showing that *hse-5(-)* partially restores aberrant pre-synaptic function in TDP-43 tg *C. elegans* (Fig 3G). We have added discussion of potential feed-back mechanisms whereby an ECM protein can signal and affect cell phosphatases and kinases. We have also included a statement of our working model for the mechanism by which *hse-5*/ GLCE protects against TDP-43.

Has GLCE showed up as a modifier of ALS in GWAS studies?
GLCE has not previously been identified as a modifier of ALS via GWAS. However, few of the previous ALS GWAS studies have been consistently replicated suggesting that many of these studies may be underpowered. We have added this information into the discussion.

Where is hse-5 expressed in C. elegans and how does this relate to the expression of mutant TDP-43?
A previous publication reported *hse-5* expression predominantly in the hypodermis and intestine [1], and this is consistent with our own observation in an P*hse-5::GFP* reporter strain (data not shown). We have added these details to the text and expanded discussion of interactions between the hypodermis, extracellular matrix, and neurons.

Were both deletion alleles of hse-5 tested?
We tested a second deletion allele of *hse-5*: *hse-5(ok2463)*. However, unlike the *hse-5(tm472)* allele used throughout this manuscript, *hse-5(ok2463)* has significant motility impairment relative to N2 (see data on right). Because this allele has additional unexpected deleterious phenotypes associated with it, we feel its use is inappropriate. We believe that precise gene deletions would be the best approach for further exploring these findings using null mutations. While such mutations do not currently exist, we plan to generate such precise deletions using CRISPR mediated genome editing. However, we believe this time consuming work beyond the scope of the current manuscript.

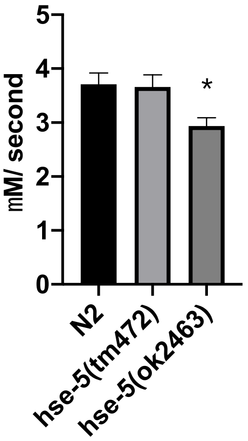

Regardless, the *hse-5* data in the manuscript includes complimentary RNAi loss of function and authentic loss of function mutation validation in *C. elegans.* In addition we show translational validation using siRNA assays of the *hse-5* homolog GLCE in mammalian cultured cells, and IHC of GLCE in human pathological tissue. Taken together, we believe the preponderance of evidence suggests *hse-5* plays a role in TDP-43 neurotoxicity.

The fact that hse-5 does not suppress neurodegeneration is unfortunate, perhaps this is the key mechanism, but at the same time maybe it is non-specific. Does hse-5 result in alternative neuronal connections that simply compensate for impaired movement in TDP-43 animals?
While we do hypothesize that alternative neuronal connections are supporting function of remaining neurons in TDP-43 tg; *hse-5(-)*, we have also added data showing *hse-5(-)* restores pre-synaptic function in TDP-43 tg (Figure 3). We have expanded our treatment of these data in the results and discussion.

Do hse-5 animals have generally improved motility?
hse-5(-) animals have similar motility to N2 (non-Tg). We have added these data as Supplemental Figure 2A.

How do they respond to aldicarb and/or levamisole?
We have tested *hse-5(-)* sensitivity to aldicarb and interestingly found a modest but significant defect in its responsiveness to aldicarb. We also tested the TDP-43 transgenic animals sensitivity to aldicarb and found a strong defect in aldicarb responsiveness. Excitingly, *hse-5(-)* was able to restore TDP-43 tg sensitivity, indicating

that loss of *hse-5* improves TDP-43 tg synaptic transmission. We have included this new data as Figure 3G and added discussion of this data in the manuscript text.

They say that RNAi candidates working in non-transgenic worms were excluded as part of the selection process, but it is possible that retesting of hse-5 RNAi in N2 worms would miss subtle effects. Thus, do hse-5 mutants have improved motility compared to N2 worms?
We have added locomotion data for *hse-5(-)* as Supplemental Figure 2A showing that it has similar motility to N2 (non-Tg).

Furthermore, a control that is missing is tests versus wild type TDP-43 transgenics. The 2010 Liachko et al. manuscript describes the WT control strains, thus these are likely the floor for the amount of suppression possible with this approach and hse-5 should be tested in these WT TDP-43 strains.
We now include data showing that *hse-5(-)* protects against wild-type TDP-43 as well (Supplemental Figure 2B).

Suppression of phenotypes associated with human disease proteins is of wide interest. Thus, it would be good to know if suppression of TDP-43 toxicity by hse-5 is specific. Does hse-5 protect against other forms of toxicity, perhaps Tau, given the role of phosphorylation in tau toxicity.
This is a good point, and to address it we have tested whether *hse-5(-)* can protect against the motility defects in *C. elegans* expressing human tau. Interestingly, we find that *hse-5(-)* does not significantly protect against either wild-type or mutant V337M tau toxicity. In fact, motility defects in these animals are slightly enhanced in the *hse-5(-)* background. We have added these data to Supplemental Figure 2.

Does hse-5 affect the expression of the TDP-43 transgenes? This would be good to know as it could change interpretation of the potential mechanism for reduced pTDP-43. Perhaps for the other 4 candidate genes as well.
We agree this is an important point, and as such, we have performed qRT-PCR to assess mRNA expression levels of the TDP-43 transgene in all suppressor mutant backgrounds. We found that none of the suppressor strains have decreased expression of the transgene. These data have been included as Supplemental Figure 1A-B.

Finally, does hse-5 affect lifespan? Also good to know in terms of modifiers of age-dependent phenotypes.
We have tested the lifespan of OH1487 *hse-5(-)* and found it is long-lived relative to N2 (non-Tg); however, it does not rescue the shortened lifespan of TDP-43 tg animals. We have added these data as Supplemental Figure 3E and Supplemental Table 2.

Minor issues

Table 2 is missing.
We apologize for this and have now included Table 2 with the resubmission materials.

p. 5 "We found that 5 of the mutants tested had decreased levels of TDP-43 protein accompanied by reduced phosphorylation (Fig. 2C-G). Interestingly, these results indicate the remaining 4 suppressor genes that improve TDP-43 motor dysfunction do so without a direct impact on TDP-43 phosphorylation or protein levels." It looks like there is more of a mix of reduced total TDP-43 versus pTDP-43. It is clear that something is happening, but perhaps a simple relative quantification would aid the figure. Also, what happened to TDP-43 in the gly-8 mutants?
We have added quantitation of immunoblots to Figure 2, and have added additional immunoblots and quantitation as Supplemental Figure 1C-F). We have not explored the mechanism of TDP-43 turnover in the *gly-8* mutant as this paper focuses primarily on *hse-5*.

Reviewer #3:
The manuscript is concise and clearly written. The introduction clearly frames the significance of the work. However, the discussion section essentially restates the results and does not offer substantial insights into any of the hits or their potential significance to the field. It would be extremely useful to discuss whether any of these genes or their homologs have been identified in the other screens mentioned by the authors. If so, what can be learned? If not, what might be unique about this tdp-43 model that facilitated their identification? Could the over-expression of mutant human tdp-43 in the background of an endogenous WT worm tdp-43 influence the results of the screen? How might this be addressed? I would also like to see more discussion of why a gene involved in (mostly) extracellular protein modification that is predicting to be localized within the secretory pathway is required for toxicity of a nucleo-cytoplasmic protein. This is not clear and is one of the most interesting conclusions from this work. There is also a lot known about the role of hse-5 in neuronal migration and axon regeneration from previous work, which is basically not discussed. Could these functions possibly explain how hse-5 might be protecting against tdp-43? Additional discussion in this area is warranted.

We agree that these are interesting points to add to the discussion. We have included details of the few genes previously identified in either ALS patient tissue or TDP-43 model organisms to the discussion. We have also expanded discussion of the *C. elegans* model, including some of the unique features that make it a useful model for studying TDP-43 phosphorylation in particular. It is unlikely that the endogenous *C. elegans tdp-1* gene is affecting the phenotype of the TDP-43 tg animals. We have previously published characterization of the TDP-43 tg strain in a *tdp-1* deletion mutant background, and found no effect of loss of *tdp-1* on the TDP-43 tg phenotypes [2]. We have also added more discussion of potential relationships between TDP-43 and ECM proteins, and included more details about previously published work studying *hse-5* in neuronal migration and axon regeneration.

I also have several major and minor concerns regarding the data as presented:
Major
1. In the screen flow chart, the authors indicate that gene knockdowns that are "different for TDP-43 tg versus non-transgenic" are discarded. Given that the phenotype being screened for is improved motility in the tdp-43(M337V) background, how would one see improved motility in a non-tg (ie wild type) background? Were any hits eliminated based on this criteria? Or was the filtering for gene knockdowns that had similar motility in WT and tdp-43(M337V)? This description needs to be clarified.
The description of motility changes in the non-Tg background is not applicable to this screen and so we have removed it from the flow chart (see revised Figure 1A).

Along these lines, it would be useful to see the motility of WT in Fig 2A and 2B.
We have added motility of N2 in Supplemental Fig. 1A, and included details of N2 motility into the figure legend of Fig. 2.

2. Figure 2 C-G, the authors show that 5 loss of function mutants, including hse-5, exhibit reduced phosphorylated tdp-43(M337V) protein levels, as well as total tdp-43(M337V) levels (although paqr mutants look to have similar total tdp but reduced phospho but the authors do not discuss this). The reduction in phospho-tdp could be due to several less interesting effects that have nothing to do with tdp-43, including transgene suppression (ie gly-8 mutants appear to have NO tdp expression) or reduced snb-1 promoter activity and subsequent reductions in tdp mRNA. Were any of these possibilities examined? This is important not only for the authors to interpret their data but also to prevent others from chasing non-existent tdp-43 toxicity mechanisms.
All three reviewers raised this important point about whether suppressors were affecting mRNA expression of the TDP-43 transgene. To address it, we have performed qRT-PCR to assess mRNA expression levels of the TDP-43 transgene in all suppressor mutant backgrounds. We found that none of the suppressor strains have decreased expression of the transgene. These data have been included as Supplemental Figure 1A-B.

3. The authors state that the results from Fig 2C-G "indicate the remaining 4 suppressor genes improve TDP-43 motor dysfunction without a direct impact on TDP-43 phosphorylation or protein levels." Given that no data were presented for these 4 mutants, there are no data in the manuscript to support this conclusion.
We now include the immunoblot and quantitation data for the suppressor genes that do not change TDP-43 protein levels as Supplemental Figure 1.

4. On pg7, para2, the authors state "Loss of C. elegans hse-5 protects against accumulation of phosphorylated TDP-43(Fig. 2C). This is not supported by the data, which show a qualitatively similar decrease in both total tdp-43 protein levels and phospho-tdp-43 levels in hse-5 mutants. Therefore, when hse-5 is lost, there is LESS tdp-43 to protect against.
We have added quantitation of independent replicate immunoblot data for all suppressor candidates (Figure 2C-G and Supplemental Figure 1) and discussion of these results in the text to facilitate interpretation of the results.

Minor
1. Why does 2A show motility data in the M337V while 2B shows it in the A315T? Have any suppressors been identified that exhibit allele-specific suppression? Is there something preventing comparisons in the same tdp-43(M337V) background, given that authors went to the trouble to make two unlinked integrated strains?
We used two different TDP-43 transgenic strains because some of the suppressor candidates were present on the same chromosome as our primary TDP-43 tg strain (chromosome IV). To assay genes on chromosome IV, we crossed these candidates to another TDP-43 tg strain with the transgene integrated on chromosome II. We have clarified the rationale behind these choices in the manuscript text and figure legend for Figure 2, and for clarity now refer to the second TDP-43 transgene used as TDP-43 tg2.

2. There are two Figure 4C panels
Fixed.

3. The PDF file that I reviewed lacked Table 2, which is referenced in the paper at least twice. Not sure if this was an author or a journal issue.

We apologize that Table 2 was missing. We have included Table 2 with the resubmission materials.

4. The reduction in GLCE protein in the frontal cortex of FTLD patients is interesting. The hypothesis that reduced GLCE expression in the FTLD patients is protective is quite provocative. If this is true, one might expect GLCE expression levels to be normal in other brain regions not undergoing neurodegeneration. Have other non-degenerating regions been examined?

We only had access to non-degenerating brain region (cerebellum) tissue from one case and one control. We have immunostained those regions for GLCE, and although GLCE expression appears to be slightly reduced, we cannot draw conclusions with such a small sample size. We have included these data here, but not in the paper due to the limitations of the sample size available. This will be interesting future work to pursue when we have access to more patient tissue.

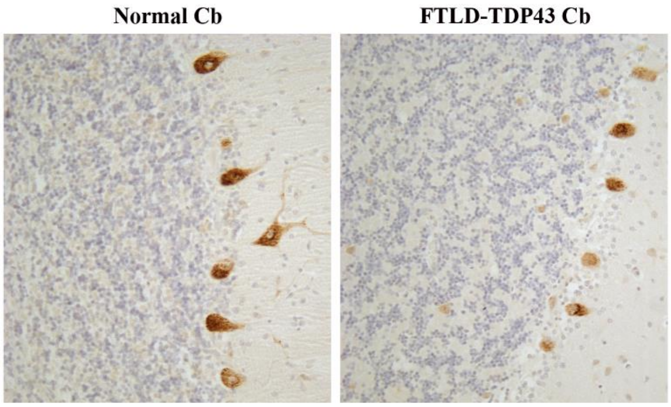

Perhaps this could be more easily addressed in worms. For example, is hse-5 expression down-regulated in tdp-43(M337V) worms?

We tested whether *hse-5* gene expression changes in our *C. elegans* model using qRT-PCR. However, we did not observe any difference in *hse-5* expression between non-Tg and TDP-43 tg worms. We have added these data to Supplemental Figure 2.

References

1. Bulow HE, Hobert O. Differential sulfations and epimerization define heparan sulfate specificity in nervous system development. Neuron. 2004;41(5):723-36. doi: 10.1016/s0896-6273(04)00084-4. PubMed PMID: 15003172.

2. Liachko NF, Guthrie CR, Kraemer BC. Phosphorylation Promotes Neurotoxicity in a Caenorhabditis elegans Model of TDP-43 Proteinopathy. J Neurosci. 2010;30(48):16208-19. Epub 2010/12/03. doi: 30/48/16208 [pii]
10.1523/JNEUROSCI.2911-10.2010. PubMed PMID: 21123567.
