# Supplementary material for: Genome wide analysis reveals heparan sulfate epimerase modulates TDP-43 proteinopathy
Source: PLoS Genet. 2019 Dec 13;15(12):e1008526. doi: 10.1371/journal.pgen.1008526 (PMC6934317; doi:10.1371/journal.pgen.1008526)
Supplement: Attachment — Submitted filename: PLOSGenetics_cover letter_resbut_10_29_19.docx.pdf [file pgen.1008526.s004.pdf]

DEPARTMENT OF VETERANS AFFAIRS
Puget Sound Health Care System
1660 South Columbian Way
Seattle, WA 98108-1595

Dr Gregory Barsh
Editor-in-Chief
and
Dr. Anne Hart
Associate Editor
PLOS Genetics

November 3, 2019

Dear Drs. Barsh and Hart:

We would like to express our gratitude for both the Editors' and Reviewers' acceptance of and enthusiasm for our manuscript entitled "Genome wide analysis reveals heparan sulfate epimerase modulates TDP-43 proteinopathy**."** We look forward to publishing in *PLOS Genetics* and are glad that our previous efforts have fully satisfied Reviewers 1 and 2. We understand the residual matters raised by Reviewer 3 and believe these are readily addressable. We appreciate the Reviewers' thoughtful consideration of our work and feel their critiques and recommendations have significantly improved our manuscript.

Below, we provide a list of the remaining issues raised by Reviewer 3 and respond to these concerns both in the written response below and by changes in the text of the manuscript where appropriate.

Thank you for your attention. Please let us know if any additional modification of the manuscript is needed. We look forward to seeing the proofs!

Sincerely,

Associate Director for Research
Geriatric Research Education and Clinical Center
Veterans Affairs Puget Sound Health Care System
Research Associate Professor, Medicine,
Psychiatry and Behavioral Sciences,
University of Washington
1660 South Columbian Way
Seattle, WA 98108
Phone 206-277-1071

Reviewer #3:

1. Were RNAi clones sequenced to confirm their identity? Neither the methods nor the results state *that they were sequence verified. Table 2 suggests that many 'hits' were not phenocopied using LOF mutants. One possibility for this is that the RNAi clone targeted a different gene. From many RNAi screens, we find that ~10% of Ahringer library RNAi clones do not correspond to the predicted gene when sequenced.*

All clones were confirmed by Sanger sequencing. We now state this in the screening methods.

2. There are still some nomenclature issues
a. Allele names in Table 2 are not italicized
b. Mutant name in Fig 2A & B, 3A,B,C,G, S1A,B,S2A-E are not italicized. Also, the allele should be listed, not (-). There is no evidence presented that these alleles are null mutants, as implied by the (-) designation

We have made both Table 2 and the stated figures conform to standard nomenclature. We appreciate the Reviewer's assertion that tm472 is not a null allele and we have no direct evidence supporting it being a null. It is certainly a strong loss of function but we cannot exclude the possibility that it encodes some truncated peptide.

3. P7 ' To confirm mRNA expression of the TDP-43 transgene in these strains…'. I believe the authors meant to say ' To determine if TDP43 mRNA expression levels were altered in these strains…'. To me, statements that aim to confirm imply that the authors are trying to prove their hypothesis is true, rather than attempting to disprove their hypothesis.

We appreciate the feedback and agree this better captures our intent and improves the manuscript.

4. The extended paragraph on hse-5 expression, mutant phenotypes, physiological roles, etc (p7-8) doesn't seem to belong in the results section and should be moved to the discussion.

While we appreciate the Reviewer's position on this, we feel that the paragraph as written provides important context for this particular hit and both emphasizes why we chose to focus on it and sets the stage for the presentation of the translational studies in Fig 4. We can certainly modify the manuscript as recommended by reviewer 3, but feel it would detract from the overall presentation. We will conform to whatever guidance given by the editors on this matter.

5. The authors refer to an interaction between hse-5/GLCE and TDP-43 (p9). Since the authors have not demonstrated that there is a physical interaction between these two proteins, they need to be more precise with this statement and indicate that they are referring to a genetic interaction.

We have clarified the language indicating this interaction is genetic rather than physcial.

6. I find the first two pages of the discussion section to be largely uninformative. It simply restates the molecular associations for many of the hits from this screen. Virtually all of that information is found in Table 1 and the discussion does not really expand on anything. On the other hand, the discussion that occurs after these sections is much improved.

We appreciate Reviewer 3's thorough digestion of Table 1. However, we believe the first section of the discussion is necessary to highlight TDP-43 suppressors with human homologs and provide additional information about them in the context of cellular function and human disease. Most of these hits are not discussed anywhere else in the text, and it is possible that the information will be of interest to the general readership. Given that the results in Table 1 are at the heart of this work, we believe they should be summarized and placed in contexts in the discussion.
